# Design and Validation of an E-Textile-Based Wearable Sock for Remote Gait and Postural Assessment

**DOI:** 10.3390/s20226691

**Published:** 2020-11-23

**Authors:** Federica Amitrano, Armando Coccia, Carlo Ricciardi, Leandro Donisi, Giuseppe Cesarelli, Edda Maria Capodaglio, Giovanni D’Addio

**Affiliations:** 1 Department of Information Technologies and Electrical Engineering, University of Naples ‘Federico II’, 80125 Naples, Italy; federica.amitrano@unina.it (F.A.); armando.coccia@unina.it (A.C.); 2 Scientific Clinical Institutes ICS Maugeri SPA SB, 27100 Pavia (PV), Italy; carloricciardi.93@gmail.com (C.R.); leandro.donisi@unina.it (L.D.); giuseppe.cesarelli@unina.it (G.C.); edda.capodaglio@icsmaugeri.it (E.M.C.); 3 Department of Advanced Biomedical Sciences, University of Naples Federico II, 80131 Naples, Italy; 4 Department of Chemical, Materials and Production Engineering, University of Naples Federico II, 80125 Naples, Italy

**Keywords:** wearable devices, e-textile, gait analysis, m-health, plantar pressure, validation, Internet of Things

## Abstract

This paper presents a new wearable e-textile based system, named SWEET Sock, for biomedical signals remote monitoring. The system includes a textile sensing sock, an electronic unit for data transmission, a custom-made Android application for real-time signal visualization, and a software desktop for advanced digital signal processing. The device allows the acquisition of angular velocities of the lower limbs and plantar pressure signals, which are postprocessed to have a complete and schematic overview of patient’s clinical status, regarding gait and postural assessment. In this work, device performances are validated by evaluating the agreement between the prototype and an optoelectronic system for gait analysis on a set of free walk acquisitions. Results show good agreement between the systems in the assessment of gait cycle time and cadence, while the presence of systematic and proportional errors are pointed out for swing and stance time parameters. Worse results were obtained in the comparison of spatial metrics. The “wearability” of the system and its comfortable use make it suitable to be used in domestic environment for the continuous remote health monitoring of de-hospitalized patients but also in the ergonomic assessment of health workers, thanks to its low invasiveness.

## 1. Introduction

The term Electronic-Textiles, or E-Textiles, refers to a wide range of studies and products that extend the usefulness and functionalities of common fabrics. The innovative feature taken by this novel application regards the embedding of digital components, such as batteries, LEDs, and, in general, electronic components, in common fabrics. Thus, through E-Textile technology, every kind of digital application can be potentially developed on a textile substrate. This attractive opportunity is bringing a revolution in the market of wearable devices, with the involvement of big companies which are trying to shift from the wearable electronic hardware to the more comfortable electronic textiles. The market of wearable technologies has a compound annual growth rate of 15.5%, with great opportunities of expansion, it is expected to reach 51.6 billion USD by 2022 (IDTechEx). E-textile is gradually covering this market, offering cheap and comfortable solution in different sectors, such as fashion, entertainment, military and defense, space exploration, health, and wellness.

Healthcare remains one of the most interesting and promising markets: e-textile features are very suitable for the development of innovative medical devices or applications that can potentially establish significant cost reductions for healthcare systems. Wearable devices for health monitoring can be easily used by patient in domestic environment and, when they are integrated in a complete communication chain, they allow smart remote monitoring with great benefits for caregivers and patient himself. E-textile sensitive fabrics can be developed to acquire and react to clinical signals detectable on body, with some interesting advantages: first, the nature of fabrics makes them the best solution to realize sensors in direct contact with the skin; second, fabrics are flexible and well adaptable to human body offering technological possibilities not available with the common electronics; and third, fabrics are cheap, comfortable, washable, and easily customizable [1]. Thus, smart biomedical clothes potentially represent an innovative tool for the continuous monitoring of vital signs, combining the function of sophisticated medical devices with the comfort and ease of use of clothing products.

Moreover, the opportunity to integrate these innovative devices in IoT networks makes possible to establish smart solution for remote health monitoring, exploring the growing field of m-health and supporting cost reduction in healthcare system by facilitating early hospital discharges. Many E-Textile solutions for health monitoring have been proposed in literature, but most of them are blocked in the research field and are not intended to flow to the pragmatic healthcare world. Regulatory issues regarding patient safety, privacy, data management [2,3], and the need of a safe degree of reliability for device performances represent the main obstacles to the large commercial diffusion of such types of devices.

This manuscript presents a prototypical system, based on an e-textile sensing sock, able to collect the angular velocities of lower limbs, using Inertial Measurement Units (IMUs), and the plantar pressures, by means of textile sensors. Our aim is to provide a wearable and portable system for the assessment of both postural and gait tasks, exploiting the recent advances in the field of e-textile, electronic and signal processing. In particular the system is intended to provide the assessment of spatio-temporal gait parameters by processing the angular velocities signals while the pressure signals will be used to assess Center of Pressure (COP) displacements during static postural tests.

Static posturography in clinical environment is usually achieved by means of commercial platform systems. These systems include a big number of sensors arranged in a matrix resulting in high spatial resolution and high accuracy [4]. However, platform systems are expensive, not portable, and require a trained technician to be used. In-shoe systems can overcome the usability limitations of platforms, enabling measurements of plantar pressure distribution within a shoe, in indoor and outdoor environments. In [5,6,7], three insoles with, respectively, 10, 4, and 3 sensors are used to measure the COP for the assessment of balance. All these applications are based on force sensing resistors (FSRs), whose hard structure can reduce the comfort for the user. Moreover, insoles create an additional layer inside the shoe, which can essentially change the distribution of plantar load of the foot compared to the natural in-shoe condition [8]. Textile pressure sensors represent an attractive solution because they improve comfort for users and their thickness ensure no distortion of plantar pressure. Several experimental custom-made smart socks, with textile pressure sensors embedded, are described in literature. Most of them are developed for the assessment of spatio-temporal gait parameters [9,10,11], while other solutions [8,12] provide for postural assessment in dynamic tasks. Nevertheless, the latter offers only a qualitative representation of pressures distribution during walking tasks. Unlike these, the proposed system uses the textile pressure sensors not for gait analysis nor for dynamic postural assessment, but pressure signals are considered and processed to provide quantitative estimation and analysis of COP displacement during static tasks.

Regarding gait assessment, we decided to exclusively exploit kinematic data collected from IMUs because plantar pressure signals would not provide significant support for the estimation of spatio-temporal parameters. IMUs are nowadays broadly used in biomedical field. These devices are light, small, and can be easily integrated in electronic circuits, so they are very suitable for wearable application. Different kinds of IMU-based medical applications are available in literature, from the activity classification [13,14,15,16,17,18] to the balance assessment [19,20], but gait analysis is the most explored [21,22]. IMUs overcome the limitations of laboratory measurements enabling the assessment of spatio-temporal gait parameters in indoor and outdoor environments. Moreover, IMUs are cheaper and more practical than full gait analysis systems, thus broadening the range of its potential users. As reported in [23], gait analysis is typically gained using the accelerometer, while the gyroscope is arguably the next most commonly used sensor. The different gait phases can be detected from angular velocities, measured by gyroscopes attached to lower limbs [24]. Accelerometers by themselves can measure angular rotation but they cannot give a good result as gyroscopes. Thus, gyroscopes are often used in fusion with accelerometer readings [25,26,27], when deployed together such as in an IMU, or alone [28,29,30,31] in the assessment of gait parameters.

There is a variety of commercially available IMU-based systems for gait analysis that are currently used in clinical environment, such as Opal by APDM or G-Sensor by BTS. They are wearable and portable systems, but they are expensive and require the presence of a technician to place sensors and carry out the acquisition using the computer software. Therefore, they cannot be used in domestic environment nor without the supervision of an expert. In contrast, our system is intended to be used in real-life conditions without any aid, as it only requires to wear socks and follow the easy steps guided by a mobile application that can be installed on the patient’s smartphone.

In this manuscript, we describe the details of prototype design and development. We also provide a validation analysis of the system concerning the assessment of spatio-temporal gait parameters deriving from IMU signals digital processing. This analysis has been obtained by performing comparative assessments with a stereophotogrammetry system for gait analysis, used in clinical environment and considered to be the gold standard in this kind of assessment.

## 2. Materials and Methods

The aim of this work is to present the novel wearable device SWEET Sock for remote health monitoring and to validate its performances in the acquisition and analysis of angular velocity signals of the lower limbs for the assessment of spatio-temporal gait parameters. The first version of this device, presented in [32], has been improved with new more efficient textile and electronic components and through the addition of a set of signal processing algorithms. In this chapter, we will present in detail the units making up the update version of the system and the materials and methods used to perform the validation analysis.

### 2.1. Wearable Device: SWEET Sock

**Figure 1:**
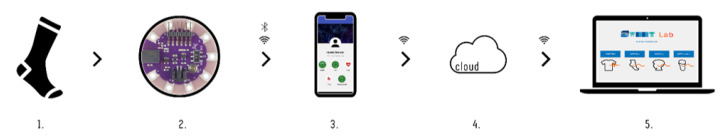
System Architecture: (**1**) SWEET Sock—Textile Unit; (**2**) SWEET Sock—Control Unit; (**3**) SWEET App; (**4**) Web Server; (**5**) SWEET Lab.

SWEET Sock is a wearable sensing device which allows the acquisition of accelerometric and pressure signals. It can be integrated in a complete system for remote health monitoring, presented in the schematic diagram in Figure 1.

The wearable sensor unit allows the acquisition of bio-signals when connected to the analogue front-end located in the electronic unit. This unit also contains a microcontroller and allows data transmission through an integrated Bluetooth Low Energy (BLE) module. A custom-made Android mobile application has been developed to receive and visualize real-time signals on a smartphone, and to upload data on a dedicated web server afterwards. This is a restricted area that is accessible after prior authentication, exclusively by authorized and appointed health professionals, who can download, analyze, and process data using the custom-made MATLAB desktop software.

In the following sections, the functional modules of the system are individually presented.

#### 2.1.1. Wearable Sensing Unit

**Figure 2:**
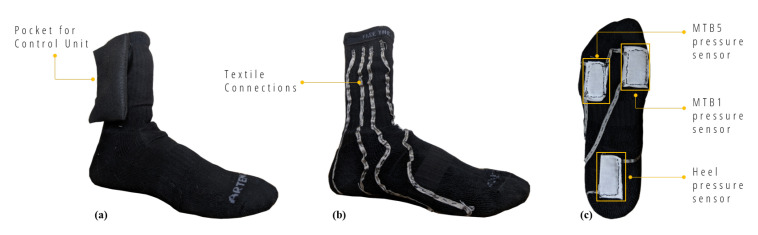
SWEET Sock sensing unit: (**a**) external view; (**b**) internal view of textile connections; (**c**) textile pressure sensors.

The wearable sensing unit consists of a commercial sports sock in which three pressure sensors, in e-textile technology, have been integrated as sensing elements in three strategic points of the foot arch. The number and placement of sensors were based on anatomical considerations: in standing position, the main force transmitted onto the foot originates at the bones of the lower leg. At the ankle, this force is divided into three smaller forces in the style of a tripod. Within the foot, one of these three forces is directly transmitted onto the calcaneus, the second one onto the first metatarsal, and the third one is distributed across the second to fifth metatarsal [33]. We therefore decided to use three pressure sensors per foot: one under the heel (HEEL), one under the first metatarsal bone (MTB1), and one under the fifth metatarsal bone (MTB5) (Figure 2c). Besides the experimental device presented in [33], also the commercial smart socks Sensoria are designed with the same number and placement of the pressure sensors. The performances of the latter in static postural assessment have been also investigated, with good results, in comparison with a stabilometric platform [34]. The use of the minimum number of sensors needed for the analysis reduces the complexity of textile design and can improve the comfort and wearability for users.

Sensors have been realized by using 2-by-4 cm sheets of EeonTex fabric, a conductive and nonwoven microfiber with piezo-resistive functionality (surface resistivity 2000 ohm/sq), offering a reduction of the electrical resistance to the application of force. Their characterization was carried out with load tests using a controlled mechanical clamp with decreasing/increasing loads [32]. The three conductive sensors have been covered by non-conductive fabric to prevent degradation by contact with the skin and are thin enough to provide postural monitoring at natural in-shoe conditions, without distortion of plantar pressure. A conductive ribbon (5 mm tick), with a resistance of less than 0.1 ohm per cm, has been used to connect sensors to the output connectors of the wearable unit. Compared to the conductive wires available on the market, the ribbon has a lower resistance (0.1 vs. 0.9 ohm per cm) and is more robust as it does not break due to stretch. The design of conductive pathways provides a placement of all connectors of the data acquisition system, represented by snap buttons, on the lateral part of the sock, which essentially improves the system usability. The textile connections have been sewn on the side of the sock avoiding, when possible, the passage under the sole of the feet, where they could be deteriorated. Connection lengths have also been minimized by studying the shortest path in order to reduce noise and interference. Figure 2 shows the complete device with its sartorial design.

#### 2.1.2. Electronic Unit

The electronic unit is a compact module containing all the electric and electronic elements to allow acquisition, digitalization, storage, and wireless transmission of the signals.

A conditioning circuit, for each conductive sensor, has been realized in order to read a voltage signal proportional to the applied force. This circuit is realized by means of a voltage divider consisting of two resistors: one of which is of known value and the other represented by the e-textile sensor. The known resistance value is fixed to 18 kohm, around which the conductive sensor resistance ranges, to reach the condition of maximal sensitivity. The IMU FLORA 9-DOF (Adafruit Inc.: New York, NY, USA) has been integrated in the electronic unit to acquire gyroscopic signal. It consists of a small electronic board mounting LSM9DS1 module, a system-in-package featuring a 3D digital linear acceleration sensor, a 3D digital angular rate sensor, and a 3D digital magnetic sensor.

A LilyPad Simblee™ BLE Board (Sparkfun Inc.: Niwot, CO, USA) has been used as the microcontroller. It provides the digitalization of pressure signals, and it is connected to Flora IMU through the I2C serial bus interface. LilyPad Simblee also allows to send data via Bluetooth Low-Energy protocol (BLE, or Bluetooth 4.0), using Simblee™ Bluetooth^®^ Smart Module integrated on the shield. BLE technology represents a perfect trade-off between energy consumption, latency, piconet size, and throughput. Its control features are implemented exploiting the ARM^®^ Cortex M0 microcontroller that can be programmed using the Arduino IDE. The control unit is programmed to sample pressure analogue signals with a sample period of 15 ms (66.7 Hz), and to receive digital data from the gyroscope with the same rate. Data are collected in 16-bytes-sized packets (2 bytes for each information: Packet, Time, x-y-z axes of the gyroscope, MTB1, MTB5, and HEEL pressure data) and real-time sent, via BLE, to the smartphone using SWEET App. Other signals deriving from IMUs (signals from accelerometer and magnetometer) are not recorded by the device because they do not provide any essential information for the planned assessments. We actually choose to implement a gyroscope-based algorithm to evaluate all spatio-temporal metrics because accelerometer signals are affected by gravity and are sensitive to sensor location [35]. When using accelerometers, it is important that they are placed in the same location each time as the signal is affected by how far from the center of rotation they are. The advantage of using a shank mounted gyroscope compared to accelerometers is that, as long as the gyroscope is recording data in the correct plane, it does not matter where on the shank the sensor is placed [36,37]. This reduction in the amount of acquired and sent data allows to improve signals sampling and sending rate.

**Figure 3:**
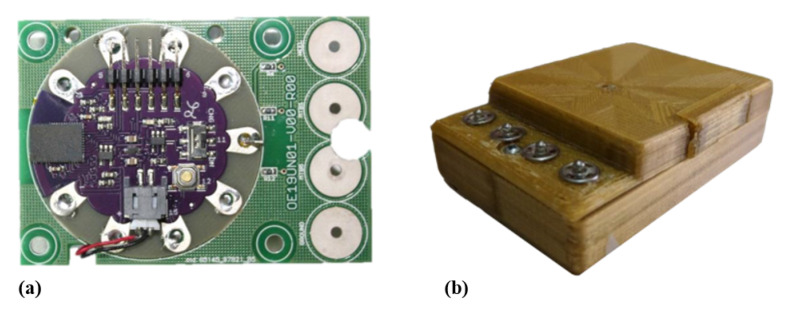
SWEET Sock ElectronicUnit: (**a**) internal electronic unit; (**b**) complete unit external view.

All modules making up the electronic unit are powered by a 190 mAh/3.7 V lithium battery, placed on the back of the same unit. The electronic unit is housed in a 3D-printed plastic case (73 mm × 52 mm × 21 mm). On the top part of the case, 4 snap buttons allow the connection to the wearable sensing unit, in order to provide the input signals for the analogue front ends. In Figure 3 the electronic unit, with its main details, is shown.

#### 2.1.3. SWEET App

**Figure 4:**
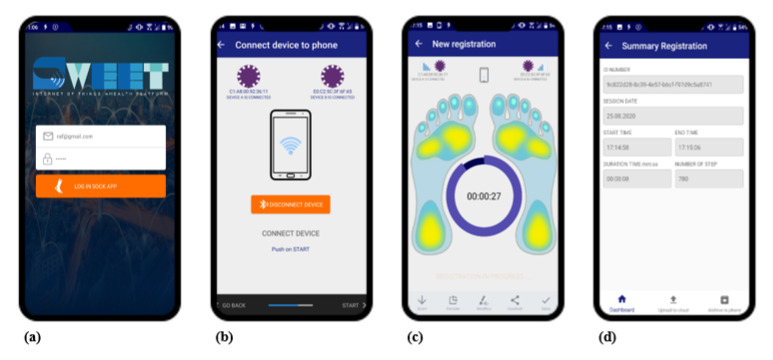
SWEET App main frames: (**a**) login; (**b**) unit connection; (**c**) signal recording; (**d**) results summary.

SWEET App is a custom-made Java language application for mobile devices requiring Android 6.0 or higher operating system and BLE technology. The application allows the smartphone to communicate and receive data coming from the electronic unit, via BLE protocol. When the application is started it is possible to associate and connect the wearable device, using its MAC address. Then, the measurement session can start, data are transferred from the electronic unit to the mobile device, which allows signals real time plotting. At the end of the session data are automatically saved in a “.csv” file, which is stored locally and can be uploaded at any time to a dedicated web server. In Figure 4 the main frames of the app are shown.

#### 2.1.4. Signal Processing Algorithms

A custom-made Matlab GUI software, named SWEET Lab, has been developed to allow signal visualization and digital processing. Health professionals have the possibility to download data from the server and analyze them using the tools offered by this software. Pressure and gyroscope signals gathered by the hardware are individually processed to respectively perform posturographic assessment and spatio-temporal gait analysis. The two types of signal were not integrated because they are used in the analysis of two separate phases: pressure signals for static postural assessment while angular velocities in dynamic walking tasks analysis.

A gyroscope-based algorithm for gait analysis has been developed. The angular velocity signals on the sagittal plane are selected and low-pass filtered with 5th order Butterworth filter (cut-off frequency 5 Hz) to reduce noise. Mid-swing, heel-strike, and toe-off events are then identified on the filtered signals for both feet, using a threshold-based algorithm [38]. The starting point of the algorithm is the identification of the time events corresponding to the mid-swing, identified as the local maximum peaks of the signal. In the next step, local minimum peaks prior and after the mid-swing point are selected as, respectively, toe-off and heel-strike time events. Starting from these gait events times, all temporal parameters of gait analysis are calculated. In Table 1, the list of temporal parameters is provided with a description clearly outlining the methods used to calculate them. Spatial parameters are assessed using a single pendulum model described in [36], where the distance from the foot to the top vertex of the rotation is modeled as equal to the height of the subject multiplied by a scaling factor. Equation (1) shows how the stride length is calculated:

(1) StrideLength(m)=S×H×2(1−cosθ)

*S* represents the scaling factor chosen equal to 0.52 [36], H represents subject height [m] and θ is the angular displacement in the sagittal plane during the stride [rad], assessed by integration of the gyroscope signal.

Plantar pressure signals collected by the sensorized socks are used to perform sway analysis, as a systematic assessment of the readiness and stability of the human body to achieve and maintain equilibrium. This analysis starts with the estimation of the center of pressure (COP), whose displacement during stand task is a meaningful parameter for a quantitative evaluation of the ability to maintain equilibrium. At each instant, COP coordinates in the medio-lateral ($X_{COP}$) and antero-posterior ($Y_{COP}$) directions have been calculated by processing raw pressure data according to the following Equation (2),

(2) $$\begin{array}{c} X_{COP}=\frac{\sum_{i=1}^{N}X_{i}P_{i}}{\sum_{i=1}^{N}P_{i}}\\ Y_{COP}=\frac{\sum_{i=1}^{N}Y_{i}P_{i}}{\sum_{i=1}^{N}P_{i}} \end{array}$$

where *N* denotes the total number of sensors, and *X* and *Y* are the sensor coordinate inside the whole foot shape area and P the pressure value. The resulting signals express COP displacement along time in the medio-lateral (ML) and antero-posterior (AP) directions, with respect to a reference point located in the middle between the feet. The mono-dimensional representations of these signals constitute the ML and AP stabilograms, while the combined bidimensional plot is referred to as statokinesigram, representing the ground projection of the COP during the stand task.

Signals are filtered with a low-pass 4th-order Butterworth digital filter with a cut-off frequency of 5 Hz [39], and then analyzed in time domain to calculate a set of parameters describing the stability of the subject during the task (Table 2) [34,40,41].

Stabilometric signals are also analyzed in frequency domain. The Matlab periodogram algorithm is used to estimate power spectral density (PSD), modified using the Hamming window. Frequency assessment is provided by means of a set of measures describing the distribution of PSD, such as peak and centroidal frequencies, band powers, and others. All the parameters assessed are listed in Table 2. The description clarifies the methods used to evaluate both spatial and frequency domain metrics starting from stabilometric signals and ground projection of the COP trajectory.

### 2.2. Validation Analysis

This manuscript presents a validation analysis concerning SWEET Sock gait assessment.

In [42], a first validation analysis was performed by comparing the raw accelerometric and plantar pressure signals acquired by the prototype with those recorded by reference systems. Following the results obtained, in this work we want to proceed the process of validation of device performances exploring the results of gait assessment, in order to carry out any possible unconformity in measurement and/or processing phases managed by the new prototype. We compared spatio-temporal gait parameters calculated by SWEET Sock with those found by an optoelectronic stereophotogrammetric system. The comparison has been carried out by means of statistical methods. This section describes the methods used for data acquisition and analysis.

#### 2.2.1. Stereophotogrammetric System for Gait Analysis

The reference system chosen for the validation analysis is SMART-DX 700 by BTS Bioengineering, an optoelectronic stereophotogrammetric system used for movement analysis. Stereophotogrammetry is usually considered a “gold standard” in gait analysis when used appropriately. The system is made of 6 infrared digital cameras, with a sensor resolution of 1.5 megapixel, an acquisition frequency from 250 fps (at maximum resolution) to 1000 fps and an accuracy lower than 0.1 mm. The recognition of body segments during movement is achieved through the use of twenty-two retro-reflective passive markers (diameter 14 mm), which are attached to subject’s skin at specific landmarks. Video data are processed on a PC workstation running SMART Clinic software, able to store and compute a set of parameters concerning kinematic (spatiotemporal parameters, joint angles) and dynamic (forces exchanged).

#### 2.2.2. Experimental Setup

**Figure 5:**
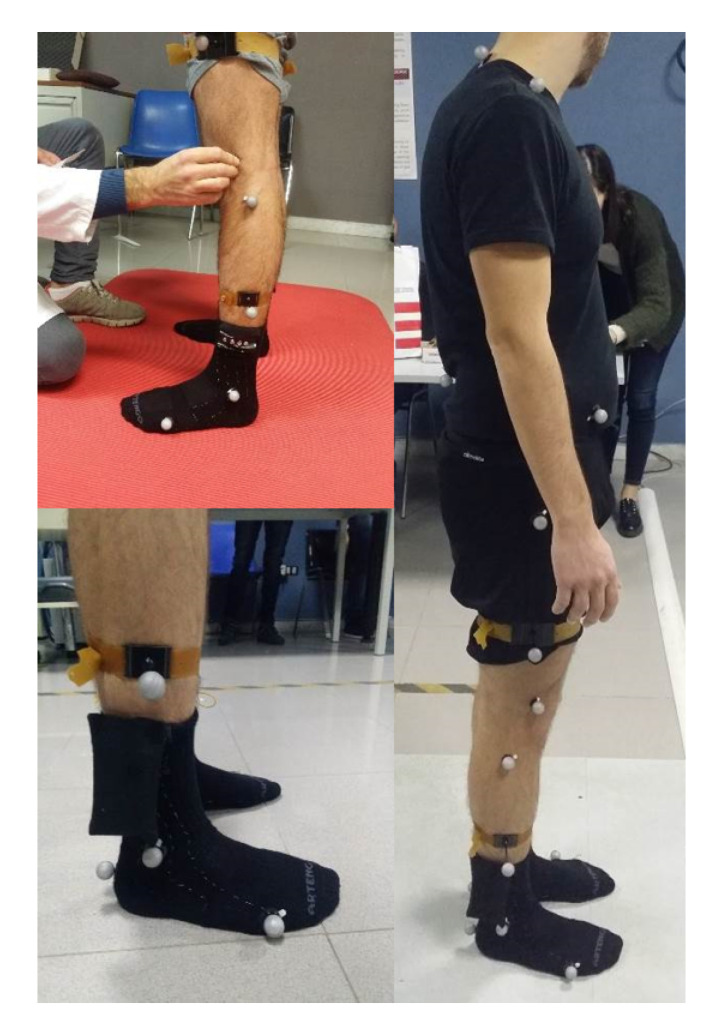
Subject equipped with both systems: SWEET Sock and reflective markers.

One-hundred-and-eight records were acquired on three healthy subjects: two males (aged 27 and 26) and one female (aged 25). Participants were free of neurological, muscular, and skeletal comorbidities affecting mobility and gait. The subject wore the sensorized socks connected to the electronic unit and was equipped with the markers of the stereophotogrammetric system, in order to perform simultaneous recording of the walking tasks with the two systems under test (Figure 5). The markers were attached to subject’s skin according to the protocol described by Davis et al. [43].

The trials involved free walking tests on a 11 m walkway in the movement analysis laboratory of University Hospital “Ruggi D’Aragona” of Salerno (Italy). Each subject was instructed to perform eight independent trials respectively at preferred, slow and fast self-selected walking speed. After that, the use of a metronome was introduced to force subjects walking at fixed normal, slow and high speed. Metronome rate was set at 100%, 67%, and 133% of the average cadence previously assessed for each subject over 5 free walking tests using the accelerometers-based gait analysis system Opal by APDM. Subjects performed four walking trials at each speed imposed by metronome. The trials were performed at different walking speed in order to obtain a dataset covering a wider range of values. Doing so, we expect a more specific characterization of the relationship existing between the two methods over all the range of measurement.

In order to validate the proposed e-textile wearable system, the gait analysis parameters obtained from this device have been compared with those obtained by the reference system. Starting from gyroscope signals measured by SWEET Sock, spatio-temporal gait parameters were computed by the custom-made MATLAB algorithms shown in the previous paragraph. The corresponding parameters assessed by the reference system were retrieved from the reports generated by SMART CLINIC software.

The following spatiotemporal parameters were considered for the benchmarking analysis; Gait Cycle Time (s), Cadence (step/min), Stance Time (s), Swing Time (s), and Step Length (m).

#### 2.2.3. Statistical Analysis

The agreement between measurements computed by the two systems—SWEET Sock and SMART-DX 700—was investigated by means of two-tailed paired *t*-test, Passing–Bablok regression, and Bland–Altman analysis. The paired *t*-test has been performed for all the parameters selected for the analysis, in its parametric or nonparametric form (Wilcoxon matched pairs signed-rank test) in according to D’Agostino–Pearson omnibus normality test result. With the paired *t*-test, the null hypothesis of no difference between the two systems in mean values of each spatio-temporal parameter was tested. A two-tail test was used and the nominal alpha level was set to 0.05 [44]. In combination with the *t*-test, the linear correlation between each pair of measurements has been assessed, using Pearson’s correlation coefficient (r). The agreement was further investigated using PB regression and BA plots, with the aim to find out any proportional or constant systematic error between the two methods of measurement.

Passing–Bablok regression is a method proposed in 1983 for testing the agreement of two sets of measurement achieved by different systems [45]. The novelties taken by this method, with respect to the standard linear regression are that it is based on nonparametric model, it is not sensitive towards outliers, and it assumes imprecision in both measurement methods and that errors in both methods have the same distribution, not necessarily normal. As quantitative outcomes, this method returns slope (proportional systematic error) and intercept (constant systematic error) of the fitting linear model. The quantitative-based rules to accept the agreement between systems are whether the confidence intervals (CI) of slope and intercept contain respectively 1 and 0 [45].

Bland–Altman analysis is a graphical method based on the plots of the differences between two measurements against their averages, and it is the most popular method used to measure agreement between two measurement systems [46]. If the differences are randomly distributed around the zero-value axis, no proportional nor systematic error is underlined by the analysis. Quantitative assessment is given through the bias, as the mean of the differences, and the limits of agreement (LoA) assessed as the bias ±1.96 times standard deviation of the differences [47,48]. If the differences between methods do not have a normal and/or symmetric distribution, LoA are considered to be between the 2.5% and 97.5% percentiles. Significant statistical errors are said to be present if the confidence interval does not contain zero value. Bland and Altman propose to accept the agreement between the methods under test if this interval contains zero value [47].

Statistical analyses were performed using R software (ver. 4.0.3).

## 3. Results

We approached the analysis of agreement between the two methods of measurement performing a paired *t*-test on all the parameters considered for the analysis. For each parameter, the values deriving from all the trials performed were considered, with no separation between subjects or walking speeds adopted. Table 3 shows mean and standard deviation values of each analyzed parameter dataset for each system of measure. The results of the two tailed paired *t*-test, with a confidence interval of 95%, are reported using a symbol in accordance with the following convention: ns *p*-value > 0.05, * *p*-value < 0.05, ** *p*-value < 0.01, *** *p*-value < 0.001, **** *p*-value < 0.0001. The hypothesis of no difference between systems was tested, so lower *p*-values suggest rejecting the accordance of systems. In the same table Pearson’s r values are reported.

**Figure 6:**
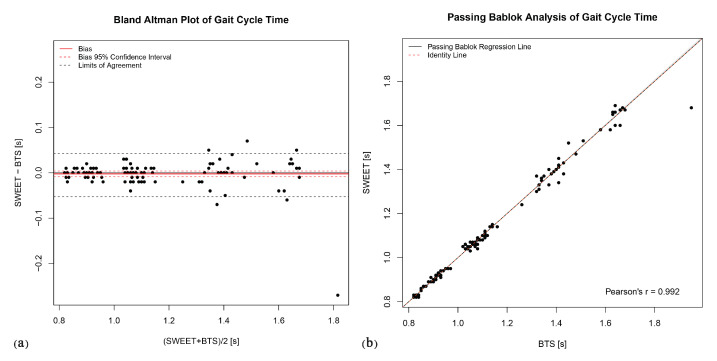
Gait cycle time: (**a**) Bland–Altman plot; (**b**) Passing–Bablok regression analysis.

**Figure 7:**
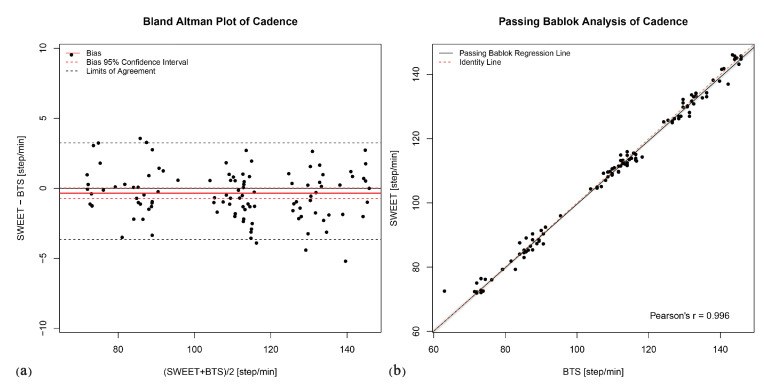
Cadence: (**a**) Bland–Altman plot; (**b**) Passing–Bablok regression analysis.

**Figure 8:**
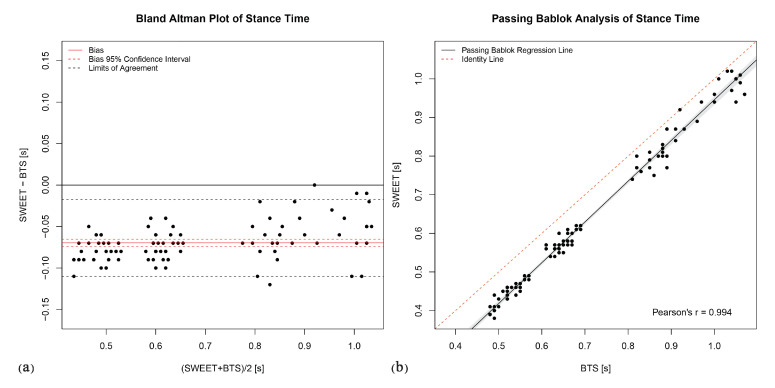
Stance Time: (**a**) Bland–Altman plot; (**b**) Passing–Bablok regression analysis.

**Figure 9:**
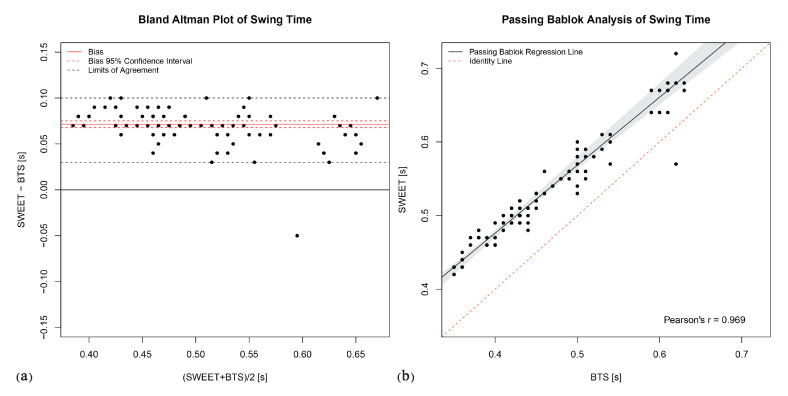
Swing Time: (**a**) Bland–Altman plot; (**b**) Passing–Bablok regression analysis.

**Figure 10:**
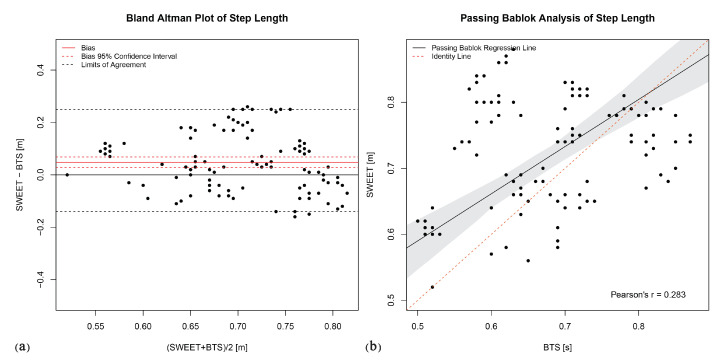
Step length: (**a**) Bland–Altman plot; (**b**) Passing–Bablok regression analysis.

The Bland–Altman analysis produces the plots shown in Figure 6a, Figure 7a, Figure 8a, Figure 9a and Figure 10a. They provide a qualitative assessment of the distribution of the differences between methods. The descriptive numeric values deriving from the analysis are reported in Table 4. The bias represents the mean of the differences between the measures computed by the systems, it is provided with the limits of its 95% CI. In the plots, biases are reported as continue red lines, while the red dashed lines represent the corresponding confidence intervals. The LoA reported in table are also shown in the graphical representations as black dashed lines. They are assessed as the 2.5 and 97.5 percentiles of differences, as they do not have a symmetric gaussian distribution.

The last analysis on data was performed using Passing–Bablok regression. In addition to the previous analyses, this analysis can reveal the presence of a trend between the measures of the two systems, thus indicating a proportional error in the tested method according to the slope of the fitting regression line. Figure 6b, Figure 7b, Figure 8b, Figure 9b and Figure 10b show the scatter plot of the dataset for each parameter, with the Passing–Bablok regression line in black. The shaded area around the regression line represents its CI, while the red dashed line corresponds to the reference identity line, to which the regression line should be tend in a scenario of perfect agreement. In the Passing–Bablok plots, Pearson’s correlation coefficient (r) is also shown because high values of r justify the choice to perform a linear regression analysis. The quantitative outcomes of Passing–Bablok analysis are reported in Table 5: slope and intercept of the regression line are listed for each parameter, along with the corresponding 95% CI limits.

## 4. Discussion

This work aims to evaluate the agreement between a novel wearable and portable device for gait analysis and the gold standard of stereo-photogrammetry system. The comparative analysis has been performed on a selected group of the principal temporal and spatial parameters assessed in gait analysis by both systems. Three different statistical methods were used to properly characterize the relationship between the measurement systems under test: paired *t*-test, Bland–Altman plots, and Passing–Bablok regression analysis.

In the assessment of the mean gait cycle time, significant agreement has been pointed out by the statistical analysis. The paired *t*-test leads to a non-significant *p*-value (*p* > 0.05), suggesting to accept the hypothesis of no difference between systems. The bias value in the Bland–Altman analysis is null (0.00 from Table 4) and the LoA are very low (in the order of few hundredths of a second). The Pearson’s correlation coefficient is very high (0.992), supporting the concept of a linear dependence between the measures, explored by means of Passing–Bablok analysis. The regression line obtained with this method coincides with the identity line (slope = 1.00, intercept = 0.00), confirming the significant agreement between the two methods in assessing gait cycle time.

**Figure 11:**
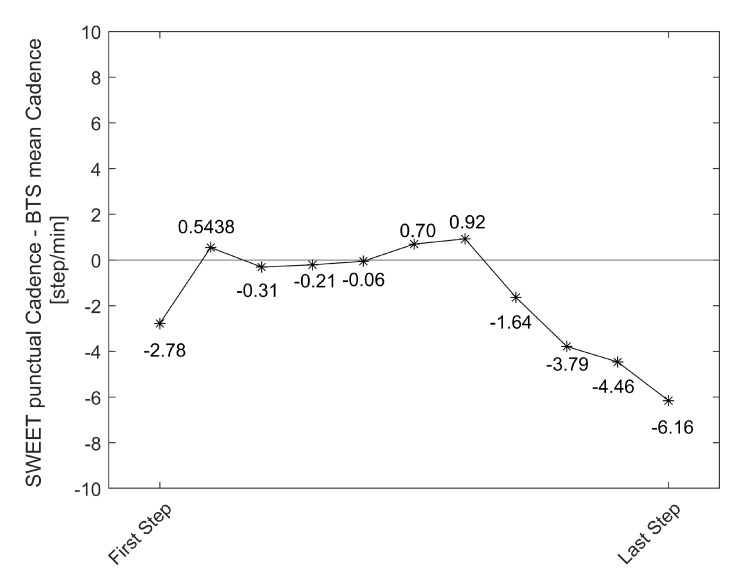
Mean difference between the punctual cadence assessed by SWEET and the mean step cadence suggested by BTS system for each step of the walking trial.

Concerning the measure of cadence, a deeper discussion is required. The *T*-test result suggests to refuse the hypothesis of absence of difference between the methods, but with low significance (0.05 < *p*-value < 0.01). The bias pointed out by Bland–Altman analysis is very low (−0.35, about 0.3% of the average value of cadence), with its 95% CI containing the zero value and limited to few units of steps per minute (−0.74 to 0.03). Passing–Bablok regression is legitimated by a high value of Pearson’s r (0.996): its slope is very close to 1 (0.99 with CI of 0.97–1.00), the intercept is different from 0 (0.74) but its CI contains this value (−0.95 to 2.38). Starting from these results and analyzing the Bland–Altman Plot in Figure 7a, we can observe that the SWEET system slightly underestimates the value of cadence compared to BTS system. Further exploring data, we identified the cause of the non-perfect agreement in the different range of steps analyzed by the two systems. The reference system SMART-DX 700 by BTS performs gait analysis on a limited range of steps, contained in the central 3 or 4 strides of the walking trial, as they are completely included in the field of view of the cameras. The detected volume cannot be extended because it is limited by the configuration of the system which considers the limited volume of the laboratory. Instead, SWEET Sock system elaborates the entire signal coming from the IMUs, removing only the first and the last steps performed to start and stop walking. The analysis of the punctual values of cadence assessed in each single step of the walking trial by SWEET Sock system clarify that in the first and last part of walking a lower step cadence is adopted. Figure 11 shows, for each step of the walking trial, the average of the differences between the punctual cadence assessed by SWEET and the mean step cadence suggested by BTS system. We can observe that in the first and last part of walking the difference is higher in absolute value, while in the middle steps it is reduced. Therefore, we can affirm that probably a better agreement would have been obtained if the same range of steps were analyzed by the two systems. We have chosen not to do so for two reasons: the first is that in SMART-DX 700 the steps to be considered in the analysis have to be chosen manually, while the signal processing of SWEET Sock is entirely automatic, and second because we have chosen not to modify the methods of analysis of SWEET system, which can provide more accurate results by taking into account the entire walking trial.

Stance and swing phase durations are complementary parameters, because they are the two parts composing the gait cycle time. Gait cycle time is defined as the time between two successive initial contacts of the same foot. Stance phase duration is the time between the initial contact and the successive terminal contact of the same foot, while swing time goes from the terminal contact to the subsequent initial contact. The complementarity of these parameters is perfectly reflected in the results of the statistical analyses. The *T*-test identified a significative statistical difference between the systems (*p*-values < 0.0001), even if a linear correlation exists in both stance and swing phase durationsas shown by Pearson’s r values, respectively 0.994 and 0.969. The Bland–Altman plots clearly show that SWEET system underestimates Stance time compared to BTS system (bias = −0.07), and therefore overestimates of the same quantity the Swing time (bias = 0.07). Passing–Bablok results confirm the presence of a systematic error in the measures: intercepts’ CIs are symmetric for the two variables and do not contain zero value (stance Cis = −0.13 to −0.09, swing Cis = 0.10 to 0.13). It also points out a proportional error proven by the fact that the slopes of the two regression lines are different from 1 (the CIs are respectively from 1.03 to 1.08 and, symmetrically, from 0.87 to 0.94). Therefore, the difference between the methods of measures is made of a constant part and a proportional part which grows when the value of the parameter is increased. The error is to be probably addressed to the wrong detection of the initial and terminal contact of the foot with the ground, made by SWEET system through the analysis of the filtered gyroscope signal in accordance to the rules illustrated by Doheny et al. in [36]. Although the gait cycle time shows very good agreement, it does not mean that the initial contacts are well identified in the signal, because they could be all translated in time of the same quantity, still resulting in good output values. To understand the error a further analysis is required on the mutual position of initial and terminal contacts identified on gyroscope signals.

The last parameter is the step length, which has been selected to investigate the performances of SWEET system in the assessment of spatial measures. Results of the statistical analysis are not very encouraging. *T*-test points out a significative statistical difference between the measures of the systems (*p* < 0.0001), that is confirmed by Bland–Altman analysis. Actually, even if the CI of bias includes the zero, it is quite wide (−0.13 to 0.25 m) for the precision required in this spatial metric. Moreover, the reduced value of Pearson’s coefficient shows that no linear correlation exists between the measures (r = 0.283), so it does not make sense to perform the Passing–Bablok regression analysis. Actually Passing–Bablok regression line in Figure 10b does not fit accurately the points, which are distributed with no detectable trend. These results allow to affirm that there is not agreement between the systems in the assessment of the step length. Moreover, in this case the cause of the error could be probably found in the processing of the gyroscope signal that lead to the assessment of the spatial parameters. The algorithm proposed in [36] is based on modeling the movement of the shank as a single pendulum, thus deriving the spatial parameters from the calculation of the angle covered by the foot during the swing phase and using geometrical consideration. A further analysis is required to understand if this model is too simplistic to represent leg swing during gait or if other aspects (device positioning, signal filtering, etc.) cause errors in the measure of spatial parameters in SWEET Sock system. Our first purpose is to try maintaining a gyroscope-based algorithm for gait assessment, by considering other more specific models proposed in literature regarding the movement of the shank during the swing phase. An example is the double segment gait model involving both shank and thigh proposed by Aminian et al. in [24]. Doing so we can avoid the use of other sensors data, such as linear accelerations, keeping the gyroscope advantages explored in the description of the electronic unit, and avoiding the reconfiguration of the entire system.

We explored scientific literature to find out and analyze other results from gait analysis systems based on similar measuring principles. Some works exist regarding validation analysis of wearable systems for gait analysis based on processing of kinematic signals. These studies address comparative analyses with clinical instruments, such as instrumented treadmill [49], force platform [50] or pressure sensitive walkway (GAITRite${}^{®}$) [35,51,52]. No works presenting a comparative analysis with the gold standard (stereophotogrammetry system) has been found. Results from the analyzed works show a common trend: temporal parameters present a better agreement than spatial metrics. Among temporal parameters, step time and GCT show the best agreement, while stance and swing phases measurements are moderately correlated with reference measures. Results presented in this article are in accordance with this trend, confirming the poor performances of IMU-based systems in assessing gait spatial metrics. Only in [35] spatial metrics show a good agreement level, that could be caused by the different placement of IMUs, placed on both feet rather than on shanks. Results from the works in [35,49] demonstrated that foot placement allow a better measurement of spatial gait parameters. However, we did not choose this placement because it can worsen the comfort and wearability of the system for users and preclude its in-shoes use.

### Comfort Assessment

In addition to the validation of technical performance, the wearability and comfort assessment was carried out in order to evaluate the acceptance of the system by final users and to identify possible areas of improvement in terms of design. To carry out this conformity assessment, an already validated methodology was used, specifically the Comfort Rating Scales (CRSs).

The wearability evaluation of a device is a multidimensional analysis: wearable devices affect the wearer in different ways. Among the effects to be taken into consideration, there are those related to comfort. When wearing something, the level of comfort can be affected by several aspects, such as device size and weight, how it affects movement, and pain.

The design of the sock has been implemented in order to achieve the greatest comfort for the user. The integrated pressure sensors are made of textile material, therefore are flexible and imperceptible on the skin. The electronic unit has also been designed to be as comfortable as possible for the user: it is light and it can be connected to the textile sock without the need to use bands. In fact, the use of the latter could cause discomfort to the user due to the presence of a narrow element tied to the limb.

In addition to physical factors, comfort may be affected by psychological responses such as embarrassment or anxiety. Consequently, Knight and Baber proposed that comfort should be measured across a number of dimensions and for such task they developed the Comfort Rating Scales (CRSs) [53].

The CRSs provide a quick and easy-to-use tool to assess the comfort of wearable devices, which attempt to gain a comprehensive assessment of the comfort status of the wearer of any item of technology by measuring comfort across the six dimensions described in Table 6. In rating perceptions of comfort, the scorer simply marks on the scale his or her level of agreement, from low (0) to high (20), with the statements made in the “description” column of Table 6. According to Knight and Baber, this range was considered large enough to elicit a range of responses that could be used for detailed analysis [53].

The three participants involved in the study were invited to fill in the CRSs to provide a judgment on their comfort. Table 6 shows the scores assigned, for each field, by the subjects involved in the study.

Although the evaluation was carried out on only three people, it provides a preliminary measure of the comfort of the prototype device. Knight et al. [54] have proposed five Wearability Levels (WLs), determined by proportioning the scales into equal parts (Table 7). The mean score of Emotion dimension is in the WL2 suggesting that users show little embarrassment in wearing the system. All the other dimensions were rated in the WL1 proving a high wearability and comfort of the device. However, to better identify the wearability level of the device and how to improve it, future analysis will aim to make a significant assessment of comfort, testing the device on a wider cohort of subjects.

## 5. Conclusions

SWEET Sock is a new wearable and portable device for the measurement and analysis of biosignals, based on textile sensors, able to perform posturographic assessment and gait analysis. In this manuscript, we presented the development of the system and we illustrated the validation analysis of the performances of the novel system in gait assessment.

The sensing unit is a textile sock in which textile sensors and bus structures are integrated, making it possible to use the system during normal daily activities, without any discomfort. The system includes a mobile app for real time visualization of the acquired signals and a software desktop for off-line plotting and digital signal processing.

The analysis of the performances of the system in gait assessment was performed by comparing the results given by the novel system with the corresponding values computed by an optoelectronic stereophotogrammetric system (SMART-DX 700 by BTS Bioengineering) in the analysis of 108 walking trials at different walking speeds. Study results show that the agreement is not confirmed for all the spatio-temporal gait parameters analyzed. In particular, gait cycle time and cadence are the two parameters presenting the best agreement, even if the latter presents a small systematic difference between the values computed by the two systems. Stance and swing phase durations present both systematic and proportional errors in the comparison between the methods. Although both errors could be removed by taking into account this misalignment, a further analysis will be performed to understand and correct the problems directly in the processing phase. Worse results are achieved in the analysis of spatial parameters’ agreement. The measures of step length provided by the two systems are not correlated. For this parameter, a further analysis is required to correct the issues in the computational process. Based on these findings, we can affirm that the novel system can be safely used in the evaluation of gait cycle time while some issues were found in the validation of the other temporal and spatial parameters. Future developments will concern the resolution of the problems encountered in this work and the execution of a similar validation analysis regarding the posturographic assessment provided by the system.

The innovative features of the system rely in the multiparametric approach in health monitoring and in its ease of use. The “wearability” of the system and its comfortable use make it very suitable to be used in domestic environment for the continuous remote health monitoring of de-hospitalized patients. The CRSs were used to assess the comfort of the wearable system. The scores provided by the subjects involved in the study, allow to assume a good level of comfort when the socks are used.

Another valid field of interest regards occupational ergonomics, related to the prevention of work-related musculoskeletal disorders (WRMSDs) in healthcare workers.

The use of SWEET Sock during working hours by nurses and therapists could help monitor postural and dynamic variables in activities most associated with exposure to biomechanical overload (i.e., frequent patient handling, pushing and pulling, awkward postures, prolonged standing, and significant sideways twisting).

The biomechanical advantage of using patient handling devices and technological aids, including exoskeletons, could be verified through the analysis of postural parameters. Gait analysis could help rethink preventive strategies aimed at work organization (for example by providing for the alternation of dynamic and static phases, and adequate recovery breaks). Last, but not least, balance analysis and COP coordinates could provide insights into the prevention of slips, trips, and falls, which are the second most common cause of injuries leading to lost working days in hospitals. The advantages combined in a minimally invasive device, together with the accuracy and reliability of the measurement, and the future opportunity of integration into IoT networks open new perspectives to increase the effectiveness of prevention and safety strategies in healthcare workers.

## Figures and Tables

**Table 1:** Spatio-temporal gait parameters.

**Temporal Measures**	
**Variable**	**Description**
Gait Cycle Time (GCT) [s]	Defined as the time between two successive heel strikes of the same foot.
Stance Time [s]	The amount of time a foot is in contact with the ground within a single gait cycle. It is the time between the heel-strike and the successive toe-off of the same foot.
Stance Phase [%]	Stance time expressed in percentage of the GCT.
Swing Time [s]	Duration of the swing phase, in which the foot is not in contact with the ground. It is calculated as the time between the toe-off and the successive heel strike of the same foot.
Swing Phase [%]	Swing time expressed in percentage of the GCT.
Single Support [%]	Part of the GCT in which a single foot is in contact with the ground. It is the time between the toe-off of the opposite foot and the successive heel-strike of the opposite foot, expressed in percentage of the GCT.
Double Support [%]	Part of the GCT in which both feet are in contact with the ground. It is the time between the heel-strike of a foot and the successive toe-off of the opposite foot, expressed in percentage of the GCT.
Cadence [steps/min]	Number of steps per minute.
**Spatial Measures**	
**Variable**	**Description**
Stride Length [m]	Distance covered during GCT.
Stride Velocity [m/s]	Defined as the ratio between Stride Length and GCT.

**Table 2:** Static postural assessment parameters.

**Time Domain Measures**	
**Variable**	**Description**
Mean COP coordinates [cm]	ML and AP mean COP displacements during time.
Mean Distance [cm]	Mean distance of COP trajectory from the center of the trajectory itself.
COP Trajectory Range [cm]	Maximum distance between 2 points of COP trajectory in ML and AP directions.
Root Mean Square (RMS) [cm]	RMS of COP trajectory. It is provided also for single ML and AP directions.
Angle form AP axis [deg]	Mean angle formed by the segments composing COP trajectory and AP direction.
Sway Path [cm]	Total length of COP trajectory, computed as the sum of distances between successive points of the trajectory.
Mean Velocity [cm/s]	Mean velocity of COP trajectory, computed as the ratio between sway path length and duration of the test.
95% Ellipse Area [cm${}^{2}$]	Area of 95% confidence ellipse encompassing the COP trajectory in transverse plane.
95% Ellipse Angle [deg]	95% confidence ellipse inclination with respect to the ML direction.
**Frequency Domain Measures**	
**Variable**	**Description**
Peak Frequency [Hz]	Peak frequency for ML and AP power spectrum.
Median Frequency [Hz]	Frequency below which the 50th percentile of total power is present.
80% Frequency [Hz]	Frequency below which the 80th percentile of total power is present.
Centroidal Frequency [Hz]	Spectral centroid of power spectrum. It indicates where the center of mass of the spectrum is located.
Band Power [cm${}^{2}$]	Power comprised in low [0.1–0.2 Hz], mid [0.2–0.3 Hz], and high [0.3–1 Hz] frequency bands, expressed as absolute and percentage values.

**Table 3:** Paired-*T* test.

Variable	SWEET	BTS	*p*-Value	Pearson’s r
	(mean ± std)	(mean ± std)	Summary ${}^{1}$	
Gait Cycle Time [s]	1.15±0.25	1.15±0.26	ns	0.992
Cadence [step/min]	109.30±21.85	109.60±22.25	*	0.996
Stance Time [s]	0.63±0.19	0.70±0.18	****	0.994
Swing Time [s]	0.52±0.07	0.45±0.08	****	0.969
Step Length [m]	0.73±0.08	0.68±0.10	****	0.283

${}^{1}$ ns *p* > 0.05, * *p* < 0.05, ** *p* < 0.01, *** *p* < 0.001, **** *p* < 0.0001.

**Table 4:** Bland–Altman analysis.

Variable	Bias	Lower Bound	Upper Bound	Lower Bound	Upper Bound
		Bias CI	Bias CI	LoA	LoA
Gait Cycle Time [s]	0.00	−0.01	0.01	−0.06	0.05
Cadence [step/min]	−0.35	−0.74	0.03	−3.83	3.26
Stance Time [s]	−0.07	−0.07	−0.06	−0.11	−0.01
Swing Time [s]	0.07	0.07	0.08	0.04	0.10
Step Length [m]	0.06	0.03	0.08	−0.13	0.25

**Table 5:** Passing–Bablok regression analysis.

Variable	Slope	Lower Bound	Upper Bound	Intercept	Lower Bound	Upper Bound
		Slope CI	Slope CI		Intercept CI	Intercept CI
Gait Cycle Time [s]	1.00	0.99	1.02	0.00	−0.02	0.02
Cadence [step/min]	0.99	0.97	1.00	0.74	−0.95	2.38
Stance Time [s]	1.06	1.03	1.08	−0.11	−0.13	−0.09
Swing Time [s]	0.90	0.87	0.94	0.12	0.10	0.13
Step Length [m]	0.70	0.52	0.95	0.25	0.08	0.36

**Table 6:** Comfort rating scales.

Title	Description	Subject 1	Subject 2	Subject 3	Mean
**Emotion**	I am worried about how I look when I wear this device. I feel tense or on edge because I am wearing the device.	7	4	7	6.0
**Attachment**	I can feel the device on my body. I can feel the device moving.	3	3	5	3.7
**Harm**	The device is causing me some harm. The device is painful to wear.	0	0	0	0.0
**Perceived change**	Wearing the device makes me feel physically different. I feel strange wearing the device.	5	0	0	1.7
**Movement**	The device affects the way I move. The device inhibits or restricts my movement.	5	2	1	2.7
**Anxiety**	I do not feel secure wearing the device.	0	0	0	0.0

**Table 7:** Wearability Levels.

Wearability Level	CRS Score	Outcome
**WL1**	0–4	System is wearable
**WL2**	5–8	System is wearable, but changes may be necessary, further investigation is needed
**WL3**	9–12	System is wearable, but changes are advised, uncomfortable
**WL4**	13–16	System is not wearable, fatiguing, very uncomfortable
**WL5**	17–20	System is not wearable, extremely stressful, and potentially harmful
